# Supplementary material for: Are carbon emissions trading and green financial instruments synergistic? -Comprehensive quantitative research based on content analysis
Source: PLoS One. 2024 Mar 7;19(3):e0298601. doi: 10.1371/journal.pone.0298601 (PMC10919601; doi:10.1371/journal.pone.0298601)
Supplement: S1 Data — (ZIP) [file pone.0298601.s001.zip › Data/地级市Z_F工作报告.zip!/║┌┴·╜¡/║╫╕┌╩╨/└┤╫╘╣1⁄2╓┌║┼í╛┬φ┐╦╩2╛▌═°í┐_2004.docx]

各位代表：

我代表鹤岗市人民政府向大会作政府工作报告，请予审议。

一、2003年工作回顾

　过去一年，我们认真贯彻落实党的十六大和十六届三中全会精神，在省委、省政府和市委的正确领导下，在市人大的法律监督和市政协的民主监督下，抢抓机遇，开拓创新，求真务实，扎实工作，克服了“非典”和农业受灾等困难，全面启动老工业基地振兴战略，继续推进“五大工程”，圆满完成了市十三届二次人代会确定的各项任务，国民经济继续保持强劲增长，国内生产总值完成88亿元，比上年增长10.7%，继续保持两位数增长，高于全省平均速度，创1998年以来的最好水平；财政收入完成7.06亿元，增长16.4%，再创历史新高；固定资产投资完成22.9亿元，增长15.2%，在全面建设小康社会的新征程中迈出了更加坚实的步伐。

--整体经济持续快速发展。以“五大工程”取得显著成效为主要标志，整体经济继续保持快速发展的良好态势。工业振兴工程再创新佳绩。工业经济速度、效益持续同步增长。鹤矿集团、鹤电公司、哈啤(鹤岗)公 司、东华水泥等骨干企业都比上年有较大幅度增长，鹤矿集团完成原煤产量1480万吨，比上 年增加84万吨，实现了补后盈利，对整体工业的牵动作用进一步增强；鹤电公司发电量比上年增加614万千瓦时，增加值比上年增长8.5%；哈啤(鹤岗)公司实现增加值4254万元、利税3 379万元，分别增长9.7%、9.5%。全市工业实现增加值21亿元，增长18.4%，实现利税3.37亿元，增长13.9%，其中地方工业实现增加值6.5亿元，增长19.7%，实现利税1.47亿元，增 长15.6%。重点技改项目实施顺利。共实施技术改造项目20项，累计完成投资5.69亿元，鹤 矿集团南山选煤厂技改工程、华隆玻璃2.5万吨玻璃瓶生产线、宝泉岭农管局200万头生猪屠宰加工、天泉酒业3000吨酒精生产线等14个技改项目竣工投产。闲置资产得到有效盘活，又有4户停产企业重新启动，盘活资产4010万元。绿色农业工程稳步推进。绿色无公害食品种植面积达到60万亩， 增长33.3%，绿色食品产量达到22.8万吨，增长14%；畜牧业发展势头强劲，奶牛存栏达到1.01万头，比上年增长23.6%；肉牛7.6万头，增长18.8%；生猪33.1万头，增长11.2%；牧 (渔)业增加值达到1.73亿元，增长13.4%。农业产业化经营快速推进。米业、豆业、乳业、猪业、菌业、薯业不断发展壮大，向规模化、专业化、优质化方向发展，成为农村 经济发展的重要牵动力量。农村税费改革成果显著，减轻农民负担目标完成率为100%。全市 农业实现增加值7亿元，增长14.9%，实现了一年受灾、一年恢复。城市化工程取得新突破。路桥建设全面告捷，投资9588万元新 、改、扩建了奋斗桥、支农桥和25条主次干道 、40条街巷网化道路。城市网化公路和通乡公路建设是历史上资金投入量和工程量最大的一年，完成了鹤大公路零公里至南风井段拓宽改造和4条通乡公路的建设。住宅建设势头不减，在建工程102万平方米，竣工56万平方米；农村康居工程开竣工10.2万平方米。新北方中学、华光商住广场、向阳综合市场等八项重点建筑工程完成了计划进度。

城市管理年活动决战决胜，市容市貌又有新变化。城市亮化、绿化、硬化又添新景观，对13 条主次干道和奋斗桥、新中心站广场进行了亮化建设和改造，安装路灯567盏、景观灯377组；对4个广场、29条道路进行了专业绿化，共植树2.4万株，其中大树进城9693棵，开展了树木冠名认养活动，筹集资金103万元用于 城市绿化；在红旗路、新鹤路、禾友路等主次干道两侧新铺装道板8万多平方米。城市净化取得新进展，完成集中并网改造锅炉30台，拆除大烟囱22座，居民小烟囱3480个，对中心区 炭火烧烤业户实行了集中经营；主次干道和背街小巷基本实现了全天候清扫保洁，垃圾日产日清，基本消灭了不开展委，城市中心区“白色垃圾”得到有效治理，城市面貌焕然一新。外向求进工程又上新台阶。对俄经贸合作快速发展。赴俄黄金开采成功中标，土地种植完成2000公顷，木制品加工项目正式投产，比罗比詹鹤岗大酒店迁址扩建完成并 投入使用；家电组装、红砖生产、人造大理石加工等项目进展顺利。实施对外经济技术合作及境外投资项目7个，为年计划的140%。口岸运行态势平稳，过货13.54万吨，过客1.18万 人次，因“非典”影响基本保持了上年的规模。全市完成进出口贸易总额1229万美元，增长 2.4倍，增幅居全省第二位，实现了对日、韩出口零的突破；实际利用外资236万美元，增长 18%。招商引资规模不断扩大，层次和水平不断提高。全市完成招商引资35.38亿元，增 长27.3%，引进项目184项。非国有牵动工程又有新发展。通过“领导包企”、“警企双百”、“银企联系”、“科企对接”四项重点工作的强力推进，民营经济发展的外部环境进一步改善，发展速度不断加快，全市民营经济新上项目86个， 投资额达3.2亿元；民营企业注册资金达到9.5亿元，比上年增长8%；实现工业增加值2.9亿 元，增长19.6%，实现税收1.13亿元，增长13%。老工业基地调整改造开始启动。根据国家实施东北地区等老工业基地振兴战略的一系列方针、政策，制定了我市《老工业基地调整改造总体规划》，确定了“建设两大基地、加快五业发展”的总体方向和主要任务，初步规划了100个项目，各专项规划及相关的项目规划初步编制完成，14个方面 的大项目专项推进机制正式运行，重点项目专项推进取得阶段性成效。采煤沉陷区治理二期工程获得国家批准，总投资12.12亿元，按国家确定的投资比例，可获得国家和省的补助资金8.48亿元；鹤岗发电公司二期工程通过可研评估论证，已呈报国务院待批；年产120万吨燃料甲醇项目已列为省重点支持项目，现正积极向上申报立项，并与美中贸易投资总商会、大连华丰集团等达成了合作 意向；鸟山煤矿建设项目已完成国家组织的评估论证，并纳入省“十五”发展规划；10万千 瓦矸石热电厂项目，省计委已批复项目建议书；鹤鸣米业年产100万吨大米精深加工及副产品综合利用项目 已通过国家发改委的初审、复审和专家答辩；甲醇助溶剂等项目的咨询论证工作已经完成。

　--社会各项事业全面进步。科技工作取得新进展。全年列入国家级计划项目2项、省级23项，我市工业园区被省政府批准为省级高新技术产业示范基地。人才和智力引进力度加大。组织了24个引智需求项目，圆满完成了海外专家“龙江行”的洽谈工作，与外国专家达成项目合作协议8项。教育工作成绩喜人。普通高考继续保持全省上游水平，升学综合指数居全省第三。教育改革取得实质性进展，在推进课程改革和学制改革的同时，深化办学体制改革，引进外资创办了双语枫叶学校。积极改善办学条件，投资1200万元对四中、十三中等六所学校进行了改造，使之向规范化学校迈进。卫生工作成效显著。抗击“非典”取得阶段性成果，实现了省里提出的“力争不发生、确保不扩散”的工作目标。农村卫生管理进一步加强，医疗卫生机构管理不断完善，预防保健工作成效明显，爱国卫生运动健康发展。人口与计划生育工作继续保持全省先进水平。人口自然增长率为0.41‰。广播电视工作稳步发展。新闻宣传有较大突破，事业建设大步推进，成功加入了省网络公司，扩大了有线网络覆盖面，有线电视频道由34个增加到39个。文化事业繁荣活跃。文艺创作成果丰硕，专业剧团演出实现了新突破，群众文化活动丰富多彩。体育工作再创佳绩。群众体育 空前活跃，竞技体育成果丰硕，参加省级以上各类比赛共获金牌39枚，其中亚洲比赛金牌3 枚，全国比赛金牌5枚，打破了3项省青少年纪录。社会治安形势进一步好转。坚持“以防为主、打防结合”的方针，巩固了严打整治斗争成果，刑事案件发案率比上年下降28.5%，市 公安局被评为全国严打整治斗争先进单位。

安全生产形势稳定。地方煤矿完成产量280万吨，百万吨死亡率为1.38，杜绝了三人以上重特大事故，连续四个季度获省“安康杯”竞赛嘉奖，创地煤安全生产历史最好水平，为保持社会稳定、促进经济发展做出了贡献。此外，外事侨务、民族宗教、民政、信访、档案、司法、国家安全、人防、边防等各项社会事业都取得了可喜成绩。

--人民生活水平进一步提高。城乡居民收入大幅度增长。城镇居民人均可支配收入达到5714元，比上年增长18.7%。农民人均纯收入达到2897元 ，增长44.6%。城乡居民储蓄存款余额达到104亿元，比年初增加13亿元，增长14.3%。人民群众生活质量持续提高。城市人均住宅建筑面积达到17.55平方米 ，比上年增加0.75平方米；农村人均住宅建筑面积达到21.12平方米，增加0.34平方米。 城市路桥等基础设施建设使人民生活进一步改善。城乡市场繁荣活跃。社会消费品零售总额达到23.5亿元， 增长8.6%。社会保障体系不断完善。实现就业和再就业24，275 人，再就业率为47.9%，城镇登记失业率为4.5%；政府出资购买公益性岗位，安置“4050” 下岗人员再就业2305人；完成再就业培训17，118人，超省下达计划43个百分点；大力筹措社会保障资金，企业基本养老保险金征缴率达到96%，发放基本养老保险金11，960万元，发放失业保障金1542万元，发放率为100%；支出医保基金2610万元；为符合条件的9.8万低 保对象发放城市居民最低生活保障金5115万元，拨付救灾、救济和低保资金到位率100%。

过去的一年，是我市经济发展、社会稳定、人民生活水平不断提高的一年，是全市人民 拼搏实干、开拓进取、各方面工作取得显著成绩的一年。成绩的取得，得益于我们解放思想，实事求是，全面贯彻“三个代表”重要思想，创造性地开展工作；得益于始终把发展作为第一要务，集中精力抓经济，正确处理改革、发展、稳定的关系，用发展的办法解决前进道路上的困难和问题；得益于坚持“稳中求进、有效增长”的经济工作方针，着力提高经济运行质量；得益于坚持区域经济一盘棋的思想，努力实现区域互动、相互促进；得益于坚持走可持续发展道路，在加快经济发展的同时，协调发展各项社会事业。

在看到成绩的同时，我们也清醒地看到在发展进程中存在的问题和困难：一是地方经济 总量小，接续替代产业尚不成熟；二是农业结构调整还不快，主导产业尚未形成经济优势，基础设施薄弱，综合产出能力低；三是对外开放程度不高，对外贸易和利用外资规模较小；四是大项目开发建设尚待加强，立市型、财源型的大项目还不多；五是就业和社会保障压力大，任务非常繁重；六是经济发展环境有待进一步改善。这些问题是制约我市经济社会发展的关键性问题，我们要采取得力措施认真加以解决。

二、2004年工作任务

　今年是我市深入贯彻落实党的十六大和十六届三中全会精神，抢抓机遇、乘势而上、加快 发展、加速振兴的关键一年，是继续推进“五大工程”，“努力快发展、全面建小康” 的重要阶段。中央振兴东北地区等老工业基地战略的实施，为我们提供了千载难逢的历史性机遇 。我市整体经济连续三年的快速增长，为我们进一步加快经济社会发展奠定了坚实的基础。只要我们把握形势，抢抓机遇，积极争取，创新工作，就一定能够开创经济和社会发展的新局面。 面对这样的形势，今年政府工作总的要求是：以邓小平理论和“三个代表”重 要思想为指导，深入贯彻党的十六大、十六届三中全会和中央经济工作会议精神，全面落实省、市委的战略部署，紧紧抓住国家振兴东北地区等老工业基地的历史性机遇，继续推进“ 五大工 程”，强化项目建设，加速结构调整，加快接续和替代产业培育，努力实现经济社会的持续、快速、协调发展。 依据这一要求，今年全市经济和社会发展的主要预期目标是：国内生产总值增长10%，财政 收入增长10%，固定资产投资增长15%，社会消费品零售总额增长8%，进出口总额增 长15%，城镇居民人均可支配收入增长8%，农民人均纯收入增长6%，城镇登记失业率控制在5%以内，人口自然增长率控制在5 ‰以内。

为确保上述目标的实现，要突出抓好十一个方面的重点工作。

(一)全面推进老工业基地调整改造，加快培育接续替代产业。

我们要紧紧抓住国家实施东北地区等老工业基地振兴战略的政策机遇，乘势而上，依托资源、区位 和生态等比较优势，大力培育和发展接续替代产业，加快经济结构的战略性调整，为我市的长远发展打下坚实的基础。同时，作为经济总量小、产业基础相对薄弱的欠发达地区，我市 在老工业基地调整改造中没有更多的优势，在竞相发展的热潮中既面临着机遇，又面对着严峻挑战，我们既要抓住机遇全力做好向上争取工作，更要立足于自身的努力谋求更大的发展，必须从实际出发，依托优势，科学定位，攻坚克难，创造性工作，挖掘自身潜力，激发内在活力，集中各方面的智慧和力量，

努力实现快发展、大发展。就具体工作而言，我市老工业基地调整改造的核心问题是发展接续替代产业，关键问题是坚定不移地实施项目牵动战略 。按照中央和省的部署，结合我市实际，我们提出了“建设能源、食品两大工业基地，加快化学工业、建材工业、林木加工业、旅游业和高新技术产业五业发展”的调整方向和发展重点，只有突出抓好大项目建设，才能如期完成这个调整改造的任务。为此，要进一步完善和强化高层次、专业化的项目开发和推进机制，强力推进已经确定的14个方面 重点项目的开发和建设。要加强高层次的项目规划、研究和引进工作，努力开发一批规模大、技术含量和附加值高的大项目。要切实加强项目前期工作，全力做好项目争取工作，力争有更多的老工业基地调整改造项目纳入省 和国家计划。要通过不懈的努力，使电厂二期、10万千瓦矸石热电厂、鸟山煤矿建设项目今年争取开工，沉陷区治理二期工程开始启动，120万吨燃料甲醇、石墨精深加工、斯达水泵、鹤鸣米业100万吨大米加工、黑龙江大桥等大项目取得实质性进展。与此同时，要按照老工业基地调整改造各专项规划的部署，全面做好各项配套政策的争取和落实工作，确保老工业基地调整改造工作取得阶段性成果。

　　(二)加快企业改革、改造步伐，继续推进“工业振兴工程”。

要以老工业基地调整改造为契机，以加快推进新型工业化为方向，立足于比较优势和后发优势，继续加快结构调整，扩大总量，盘活存量。一要以改造增动力，重点抓好项目 建设。要把企业技术改造作为重中之重的工作来抓，坚定不移地实施项目牵动战略，集中力量抓好21个重点项目的实施。要抓好哈啤(鹤岗)公司10万吨扩产改造、 鹤矿集团信息化工程、完达山集团宝泉岭公司日处理300吨鲜奶乳制品、萝北1.2万千瓦热电 厂等6个续建项目的建设，促其尽快投产、达产、达效，形成新的经济增长点。要抓好鹤矿集团鑫塔水泥生产线扩产改造、浙江银河药业(鹤岗)公司谷维素等系列产品开发、龙宝药业抗艾滋病药物、华欣纸业特种纸、热力公司4.8万千瓦热电联产、鲲鹏焦化公司30万吨焦炭等15个新开工项目的建设，确保完成计划进度。二要以改革增活力，进一步完善企业经营机制。要完善企业法人治理结构，按照《公司法》的要求，认真规范股东会、董事会、监事会的职能；继续深化企业人事、劳动和分配制度改革，充分调动企业经营者、管理者和员工的积极性，真正建立起适应市场经济要求、管理科学的企业经营机制。要重点抓好地方工业骨干企业内部运行机制的配套改革；全力支持中省直企业深化改革，支持鹤矿集团做好公司上市和部分煤矿破产改造工作，积极争取、落实国家的相关政策，使企业轻装上阵，提高市场竞争力。三要以园区建设促调整，加快培育新兴产业。要进一步完善工业园区总体规划，制定优惠政策，加快交通、排水、供电等基础设施和配套设施建设，增强园区的吸引力，争取再入驻3-5户企业，使园区粗具规模，为培育新兴产业奠定基础。四要以调控促发展，强化工业经济的运行监控。要突出抓好重点骨干企业的运行，加大宏观调控力度，围绕资金、运输、电力等重要生产要素，强化沟通协调，使重点骨干企业继续保持良好的运行态势，成为工业经济快速发展的重要支撑。要突出抓好停产企业的启动工作，进一步盘活闲置资产，扩充经济总量，推动工业经济健康发展。经过努力，确保工业经济速度和效益分别增长15%以上，充分发挥支撑经济全局的重要作用。

(三)大力发展畜牧业，提高产业化水平，继续推进“绿色农业工程”。

要围绕农业发展、农村稳定、农民增收，加快农业和农村经济结构的战略性调整。一要推进畜牧业大发展，大力实施“主辅换位”。要以“两牛一猪”为重点，加快畜牧业发展步伐。要落实好支持畜牧业发展的政策措施，不断加大对畜牧业的投入，扩大饲养规模，提高饲养水平。全市奶牛存栏要达到1.5万头，增长42%；肉牛饲养量达到8.2万头，增长8.6%；生猪饲养量达到43万 头，增长29.6%，畜牧业总产值、增加值占农业经济的比重分别达到38.6%和29%；实现牧业人均纯收入1073元，增长12.8%。要切实加强良种繁育和防疫灭病工作，落实好高致病性禽流感和口蹄疫等动物疫病防治措施，努力做到“力争不出现，确保不蔓延，人间不感染 ”。二要加快龙头企业建设，加速推进产业化经营。要以基地建设为基础，以市场开拓、项目建设为重点，加快龙头企业培育，加快农业产业化进程。要在不断扩大基地生产规模的同时，集中力量抓好龙头企业建设，本着资源、资金、技术、人才集中的原则，扶强扶壮一批龙头企业，招商引资建设一批龙头企业，引导民营经济大户发展一批龙头企业，推动和促进乳业、米业、豆业、猪业、薯业、菌业等产业的快速发展，带动农户3万户、基地145万亩，分别增长10%。三要推动粮食生产上水平，大力发展质量效益型农业。继续坚持打绿色牌、走特色路的发展战略，重点发展绿色无公害食品，绿色农产品种植面积要达到70万亩，增长17%。鼓励和引导广大农民走“优化结构、提高单产、改善品质、降低成本、增加效益”的发展路子，全面搞好优质粮食生产。要面向市场需求，调整粮食种植结构和品种结构，大力发展专用品种生产，积极推广模式化栽培技术，提高单位资源产出率。深入实施良种化工程和优质专用化生产，良种覆盖率和优质品率要分别达到98%和70%以上。四要加快农村富余劳动力转移，拓宽农民增收渠道。抓好用工信息收集、市场对接和农民工培训等工作，引导和推动农民外出务工，增加收入。五要加强农村基础设施建设。重点抓好通乡、通村公路建设，完成绥滨至名山段、石灰窑至兴东段、鹤岗至新华段三条通乡公路162公里和192公里通村公路建设任务。六要巩固农村税费改革成果。减轻农民负担目标完成率保持在100%。

通过采取上述措施，确保农业增加值达到7.5亿元，增长7.1%。

(四)保持建设规模，强化城市管理，继续推进“城市化工程”。

坚持“打造精品、提高品位、完善功能、便利百姓，重在雪中送炭、兼顾锦上添花”的工作思路，保持城市规划、建设、管理的良好势头。一要继续推进“518”工程。住 宅建设开竣工面积继续保持50万平方米，建设10万平方米康居工程；完成振兴广场建设；新、改、扩建振兴路、哈尔滨大街、西内环路、南工地路、哈萝公路铁东至新一段等10条城市道路；建设三中教学楼和图书馆、职 教中心教学楼、振兴花园美食城、兴山集贸市场等八大公益福利、商服设施。二要推进“五化建设”，实施“十小工程”。推进绿化、硬化、亮化、净化、美化“五化建设”，实施小街道、小道板、小垃圾箱、小果皮箱、小型移动公厕、小绿地、小市场、小停车场、小景观、小文明楼道等“十小工程”，完成义务植树30万株、专业植树1.6万株、大树进城6000棵，育 草花苗20万株，铺设草坪2000平方米；重点完成兴汇路和新世纪广场、文化广场、人民广场周边道板、边石更换铺装工作，督促临街单位完成门前的硬化铺装；完成振兴路、步行街等20余条道路的路灯安装，完成东内环至“三金之城”、时代广场至“飞鹿迎宾 ”和 振兴路的景观灯安装工作。三要继续加大城市管理力度。巩固“城市管理年”活动取得的成果，继续加强城市规划、环境卫生、公用设施、城市绿化、城市物业、居民区环境、烟尘治理、道路畅通、文明施工“九项管理”，建立长效机制，做到常抓不懈。四要强化经营城市的理念。针对城市建设资金紧张的实际，积极采取市场化运作方式，变现存量，扩大增量，走以城养城、以城 建城、以城兴城的新路子，力争在土地经营、市政公用设施特许经营、人防三期工程BOT融 资、灯箱广告经营权拍卖等方面取得新突破。五要加快培育旅游产业。按照高起点规划、高标准建设、高效能管理、高效益经营的总体思路和要求，推进重点旅游项目建设。尽最大努力争取国家和省的支持，多方筹集资金，新、改、扩建细鳞河自然保护区、将军石山庄、鹤北联营红松母树林、名山岛风景区、龙江三峡国家森林公园以道路为重点的旅游基础设施，完成萝北凤凰山滑雪场、松鹤滑雪场二期工程。开通和打造鹤岗至名山岛、龙江三峡的东部旅游线、至松鹤公园的西部旅游线、至将军石山庄的南部旅游线、至细鳞河、十里河国家森林公园风景区的北部旅游线等四条特色精品旅游线路和旅游景点，完成细鳞河、名山岛风景区国家AAA级旅游区申报工作。对市内三个公园进行总体规划和分期改造。积极宣传促销我市旅游资源、产品和纪念品，在哈巴开办国际旅行社，努力开拓域内和域外入境旅游市场，推动我市旅游产业的快速发展。要加大生态市建设力度，努力改善生态环境。

(五)扩大对外经贸合作，提高利用外资水平，继续推进“外向求进工程”。

我市外经贸工作的突出问题是规模小、水平低，因此，要以地处对俄开放前沿的区位优势为依托，以充分开发利用俄方资源和市场为重点，大力推进对外经济技术合作向宽领域、深层次发展。一要不断扩大对俄经贸合作。要抓住国家振兴老工业基地的有利时机，千方百计在对俄经贸合作上实现突破，在合作项目上从种植、养殖、采伐、餐饮向服装加工、家电组装、医疗器械生产、房地产开发、黄金开采、兴建市场等纵深领域拓展，力争完成5个对俄经贸合作项目。二要大力拓展国际贸易。要整合资源，利用我市华盛、华祥公司的出口渠道，加强与日、韩客商 的合作，共 同开拓日、韩市场。同时，通过参会参展、为企业请权办证，积极开拓东南亚、美国等市场，全年进出口总额要达到1413万美元，增长15%。三要不断完善口岸功能。要完善口岸设施，增强口岸功能，提高服务水平，优化口岸环境，力争口岸过货15万吨，增长10.8%，口岸过客1.33万人次，增长10%。四要认真做好利用外资工作。要把利用外资工作的重点放在“两大基地、五大产业”的发展上，放在老工业基地调整改造的项目开发和建设上，放宽视野，拓宽渠道，努力在日、韩等国家和港、澳、台地区的外资利用上寻求突破，力争实现利用外资100万美元。

(六)加快民营经济战略升级，继续推进“非国有牵动工程”。

要以大力发展“三型”企业为突破口，在大户培育、综合服务等方面加大力度，推进民营经济战略升级。一要抓“三型”企业发展，不断提高质量。引导民营企业积极参与老工业基地调整改造，在食品工业、林木深加工业和新型建材工业等产业上发展生产加工型企业。引导民营企业向对俄经贸合作领域延伸，针对俄方市场需求，生产适销对路的对俄出口产品，发展外向型企业。引导民营企业开发科技含量高的新产品，引进先进技术和工艺，丰富企业的科技内涵，发展科技型企业。二要抓大户培育，不断扩大规模。对营业收入超百万元的民营企业设立大户档案，跟踪问效，及时掌握发展动态。组织各方面专家成立专家小组，为民营大户提供生产经营和技术咨询服务，帮助解决实际问题。按照企业营业收入、税收增长幅度、安置就业人员数量等指标对民营大户进行星级考核，在资金、项目、包扶、保护等方面对星级企业给予重点扶持。三要抓综合服务，不断提升档次。充分发挥鹤岗私营经济网站和民营经济服务热线作用，为民营企业提供最新的国家、省、市政策、项目、科技、人才等信息、咨询服务。对投资500万元以上的“ 三型”企业项目，实行从立项、审批到正常生产的“一条龙”服务。引导民营企业与大专院校、科研院所建立广泛联系，增强科技创新能力。要强化培训，努力提高民营企业的整体素质。要强力推进“领导包企、警企双百、科企联系、银企对接”四项重点工作，充分发挥综合办公室的检查、协调和督办作用，及时通报工作进展情况，严格考核，兑现奖惩，切实把这四项重点工作抓出成效。全年民营经济要实现工业增加值3.4亿元，增长18%；上缴税金1.24亿元，增长13%；“三型”企业增加30户。

　(七)大力招商引资，积极向上争取，为经济社会发展提供资金保障。

　资金是经济和社会发展的必要保障，也是长期制约我市发展的重要因素。因此 ，要广开资金筹措渠道，最大程度地满足经济社会发展的需求。一要以招商引资年活动为契机，进一步加大招商引资工作力度。今年是全省招商引资年，我们要认真研究新形势下国内外客商的投资新热点，尽快建立成本低、效益好的招商引资工作体系，确保招商引资年活动收到实效。要进一步明确招商引资工作的重点和方向。在招商区域上，要把重点放在沿海发达地区和专业优势较为突出的省份，注重拓展与国内外友好城市的合作成果，重点在经济技术合作上寻求突破，取得实效。在招商引资的主攻方向上，要牢牢抓住国内外大型企业参与东北老工业基地调整改造的机遇，力争在引进大企业、大项目上有较大突破。在招商引资的途径上，要创新招商引资机制和方式。全面落实招商引资责任制，制定招商引资奖励措施，把对领导班子和领导干部在招商引资中的政绩考核与任用、奖励有机结合起来，形成有效的激励和约束机制。要制定优势和发展共享的政策，鼓励、支持区域内各部门开展形式多样的专业招商活动，形成区域联动、资源共享、优势互补、利益均沾、协力招商的新机制。在招商方式上，采取借助国家有关部门力量联合招商、利用中介机构委托招商、发挥各类驻外机构作用定向招商、依托工业园区和农业生态示范园区载体招商等措施，形成招商方式多元化。要注重发挥外来投资者协会、市商会和各行业协会等组织的作用，实施以商招商。全市招商引资到位资金力争达到40亿元，比上年增长15%。二要抢抓机遇，全力做好向上争取工作。要密切关注和把握国家和省的政策动向，积极抢抓机遇，加大跑省进京力度，紧紧围绕老工业基地调整改造，尽最大努力争取 上级的支持，争取更多的财源建设和事业发展的可用财力。同时，要支持金融部门增加存款，积极投放信贷资金，更好地为企业生产经营和重大项目建设服务。要积极引导民间 资本向经济领域流动，更好地满足项目建设和经济社会发展的需求。

(八)深入实施“科教兴市”和“人才兴业”战略，为经济社会发展提供智力支持。

一要大力促进科技进步和科技创新。鼓励、引导、支持企业加大科 技创新的资金投入，以发展高新技术为手段，以产学研结合为依托，以高新技术开发、引进和应用为载体，高起点、高标准地实施一批科技含量高、经济效益好的项目，增强市场竞争力。重点支持华欣纸业“高档防锈纸”、龙宝药业“泰诺夫韦”、哈星药业“降脂减肥胶囊 ”等高新技术项目，争取国家和省的配套支持。二要全面引进和培养人才。要根据我市加快发展的客观需求，立足当前，放眼长远，进一步加强人才工作。要大力推进人才队伍建设，培养急需的各类人才。积极推进人才培训的市场化、产业化、社会化，以开展职业技术教育为重点，搞好委托培训、联合办学，培养地方经济建设急需的各类实用人才。要加强引才引智工作。围绕我市老工业基地调整改造、发展畜牧业、发展民营经济等实际需求，加强国内外引智工作，扩大与大专院校、科研单位和外国专家组织的技术商务信息联系，为企业提供智力服务。要营造“尊重知识、尊重人才”的良好社会氛围，进一步完善人才引进和使用的政策，创建良好的社会环境，更多地吸引人才，有效地用好人才，为我市的经济社会发展提供智力保证。 

(九)突出抓好环境建设，继续实施“环境优化”战略。

今年是全省的改善环境年，我们要以这次活动为切入点，突出抓好环境建设。要通过加快转变职能，全面提高施政水平，努力创建行政成本低、公共服务好、市场秩序优、诚信程度高的发展环境。一要创建优良的舆论环境。要把舆论环境建设作为改善经济发展环境的首要任务来抓，利用各种宣传方式，广泛宣传，大造声势，在全社会形成“招商引资是能人，破坏环境是罪人”的新风尚，在全市营造一个“人人是环境、人人有责任”的舆论氛围。二要创建优良的服务环境。各级政府和各部门要加快由“审批型”向“服务型”转变，增强服务意识，强化服务功能。要深化办事制度改革，大力推行“阳光政务”，提高政务公开水平。要认真受理企业和投资者的投诉、申请和建议，为企业和投资者提供事前、事中、事后全过程服务。要继续推行“首问责任制、服务承诺制、限时办结制、失职追究制、否定报备制、无偿代办制”等六项制度，规范办事程序，提高办事效率。三要创建优良的法制环境。努力维护市场经济秩序，严厉打击侵犯企业经营者和外来投资者合法利益的违法犯罪行为。进一步规范行政执法 ，认真解决执法不公和徇私枉法等问题，对侵犯企业和外来投资者合法利益的违法犯罪行为要 严厉打击。对执法不公、野蛮执法、徇私枉法、办人情案，干扰、破坏经济发展环境的问题，发现一起严肃查处一起。四要创建优良的政策环境。创造性地执行中央和省的各项优惠政策，对我市改善经济发展环境的优惠政策更要用足、用好、用活。要不断研究市场经济条件下出现的新情况、新问题，及时制定和出台新的政策规 定，做到地方能定的放到最宽，外地有的要科学借鉴，外地没有的要大胆尝试，用优惠的政策吸引客商 。对制约经济发展的政策规定该废除的废除，该调整的调整。五要创建优良的诚信环境。全面履行政府承诺，增强政府决策的可行性、可靠性、可操作性，做到言必信、行必果，确保能够贯彻实施，提升政府形象。要全力抓好社会信用体系建设，制定企业信用评价和监督标准，建立企业信用档案和信用网络，引导企业诚信经营、依法纳税；建立中介机构资信评价体系，评定信用等级，规范经营行为，努力营造诚实守信、履约践诺的诚信环境。

　(十)切实关心人民生活，努力维护社会稳定。

　一要加强社会保障体系建设。采取多种有效措施筹集社会保障资金，企业基本 养老保险金征缴率要达到90%以上，拨付救灾、救济和低保资金到位率要达到100%，符合条 件的城镇最低生活保障对象9.8万人，继续保持养老保险金、失业保障金和城市居民最低生活保障金的按时足额发放，全面实现社会保障管理服务社会化。二要多渠道、多途径扩大就业。积极支持和发展有技术含量的劳动密集型产业，发挥中小企业和民营经济在吸纳劳动力就业方面的重要作用，以项目开发、民营经济发展和城市基础设施建设带动就业，以劳务输出为载体扩大就业，以政府出资购买公益性岗位增加就业，以落实好各项再就业政策促进就业，力争实现就业和再就业2万人，再就业率达到40%以上。三要加强社会治安综合治理。继续保持严打的高压态势，强化治安防控体系建设，实行群防群治，搞好社会治安综合治理，为广大市民提供一个安全的治安环境。四要加大信访工作力度。认真落实谁主管、谁负责和“一把手”负总责的信访工作责任制，加强信访基础性工作，努力把矛盾化解在基层。要建立和完善信访工作的长效机制，认真听取群众呼声，全面掌握社情民意，及时解决群众在生产、生活中遇到的实际困难。五要强化市场监管。大力整顿和规范市场秩序，严厉打击各种欺行霸市和损害消费者利益的行为，继续实施食品、药品放心工程，维护广大市民的合法权益。六要全力抓好安全工作。坚持预防为主的安全工作方针，全面落实安全工作责任制，进一步加大安全生产和交通、消防、人员密集场所的安全专项整治力度，认真开展专项检查，消除各类事故隐患，地方煤矿力争不发生三人以上的重特大事故，继续保持安全生产的良好态势，确保安全工作万无一失。

 (十一)促进经济与社会协调发展，推动社会事业全面进步。

 教育工作要继续深化改革，全面实施素质教育，加快高中阶段教育进程；不断 提高教育教学质量，使整体水平保持在全省前列；积极改善教学条件，发展特色优质学校，加快数字化学校建设，使80%的学校达到市 级规范化标准；继续加大高等教育和职业教育工作力度，整合教育资源，发展职业技术教育，做好创办高等职业教育学院的前期准备工作。卫生工作要加强公共卫生体系建设，提高处理突发公共卫生事件的能力；加强卫生执法工作，提高传染病防治水平，使传染病发病率比上年下降3%；巩固迎检成果，推动爱国卫生运动深入开展；加强农村卫生工作，提高乡(镇)卫生院的综合医疗水平体育工作要全面实施《全民健身计划纲要》和《奥运争光计划纲4要》，以提高全市人民身体素质 为目标，开展全民体质测试工作，积极开展丰富多彩的群众体育活动，全面做好备战省十一届 运动会的各项工作。文化工作要深化文化体制改革，加快文化设施建设，繁荣文艺创作和舞台，打造艺术精品和名牌，广泛开展群众文化活动，丰富人民群众生活，发展壮大文化产业。计划生育工作要加强综合治理，开展好以技术服务为重点的“优质服务年”活动，人口出生率控制在7.85‰以下，计划生育率达到97%以上， 为经济和社会发展创造 良好的人口环境。广播电视工作要加快设备更新步伐，积极创办人民群众喜闻乐见、丰富多彩的广播电视节目，努力扩大网络覆盖面，让更多的人民群众享受到高质量的电视服务。此外，民族宗教、外事侨务、民政、档案、司法、边防、人防、国家安全等各项社会事业都要取得新发展。

 三、进一步加强政府自身建设

 新的形势和任务对政府工作提出了新的要求，要顺应新形势、完成新任务，必 须坚持建设“学习型、责任型、效能型、服务型、廉洁型”政府的总体方向，不断加强自身建设，努力改进工作作风，切实提高施政水平，带领全市人民在实施老工业基地振兴战略和全面建设小康社会的新征程中取得新的胜利。

(一)实践执政为民的宗旨。政府的根本宗旨是全心全意为人民服务，必须始终把人民群众的安危冷暖挂在心上，把群众富裕作为第一目标，把群众呼声作为第一信号，把群众满意作为第一标准，切实为人民群众谋利益。一要突出加快发展的思想。欠发达地区的实际要求我们必须把加快经济发展作为政府工作的核心，作为执政兴市的第一要务，聚精会神搞建设，一心一意谋发展，不断增强综合市力，跟上全国、全省快速发展的步伐。随着全面建设小康社会进程的加快、老工业基地调整改造的深入，我们必须与时俱进、开拓进取，不断学习新知识、研究 新情况、解决新问题，进一步丰富、完善经济发展思路，勇于尝试推进资源型城市转型的有效办法，探索适应鹤岗市情的发展之路。二要强化以民为本的理念。坚持一切为了群众、一切依靠群众，全力为人民群众做实事、做好事，凡是涉及群众切实利益和实际困难的事，再小也要竭尽全力去办，真正做到利为民所谋。既要通过发展经济从根本上提高人民群众的生活水平，实现好、维护好、发展好最广大人民群众的根本利益，又要切实解决好困扰人民群众生产生活的现实问题。在努力 实现全市人民富足、富裕的同时，要特别关注下岗职工、贫困农民等弱势群体的疾苦，尽心竭力地为他们排忧解难。当前的重点是深入实施好“十万富民行动”和“十项利民行动”，开展好“五访五问活 动”，访贫困户问衣食冷暖、访温饱户问发展需求、访小康户问致富经验、访意见户问事情原委、访先进户问富民之策，竭尽全力致富一方人民。三要遵守廉洁从政的准则。要不断加强和改进党风廉政建设，认真落实廉政建设责任制，严肃查处违法、违纪案件，深入开展纠风和专项整治活动；要加强思想政治教育，推行述廉、评廉、考廉制度，建立健全教育、监督、质询、评议、重大事项报告等机制，筑牢思想和制度防线；要增强各级领导干部拒腐防变的能力，做到清正廉洁、克己奉公，自觉遵守各项纪律规定，健全正确行使权力的制约机制，使各级领导干部以廉洁从政的形象取信于民。

 (二)强化求真务实的作风。各级政府和政府各部门必须进一步改进工作作风，求真务实，真抓实干，以作风的大转变推动事业的大发展。一要突出工作重点。抓发展、搞建设，必须要抓住主要矛盾，抓住关键问题，做到重点突出、目标明确。从全市看，就是要把老工业基地调整改造作为全部工作的主线，把思想和行动统一到项目建设、招商引资、工业调整、农业发展、改善环境 等核心工作上来，集中全力培育和发展接续替代产业，加快资源型城市经济转型步伐。对于重点工作，要科学制定实施方案，明确目标、任务、完成时限、相关责任和奖惩措施，确保各项工作的有效推进和落实。二要提高工作效率。转变政府职能，改进工作作风，关键是提高工作效率，做到言必行、行必果，说干就干、干就干好。要树立能快则快、快中求好、好中求精的观念，对于事关全局的重点工作必须科学谋划、周密部署、强力推进、务求实效。各级领导干部要亲临一线，靠前指挥，以雷厉风行的工作作风和快速推进的工作节奏，加快工作运转，高质量、高效率地做好每项工作。三要健全工作机制。要进一步修订和完善指标考核体系，健全重点工作领导分工责任制，严格实行主要领导、分管领导和工作人员三级负责制，把工作责任落实到每一级、每个人，切实做到件件有着落、事事有回音，不达目的决不罢休。要进一步建立健全政府效能评议机制，大力推行责任追究制度，着力构筑领导督查、部门督查、社会监督三位一体的督查体系，认真进行考核，严格兑现奖惩，确保重点工作的顺利推进和各项任务的圆满完成。 

(三)坚持依法行政的准则。依法行政是政府行使权力和实施管理的基本准则，是规范和约束行政行为、提高行政管理水平、改进政府工作的重要前提。一要切实转变政府职能。按照职权法定、权责一致的原则，进一步规范行政执法主体，理顺各级政府和部门之间的职责权限，减少职能交叉，增强服务功能，提高工作效率。要进一步深化行政审批制度改革，科学确定审批事项，规范审批行为。对已经取消的审批事项要加强监督检查，确保落实到位；对于保留的审批项目，要减少审批环节，限定审批权限。要按照分类、脱钩、搞活、放权的原则，积极推进事业单位机构改革。要积极探索向社区转移社会管理职能的新路子，适时划分事权、下移工作重点，巩固社会保障、治安、卫生进入社区的成果，适时推进就业、城管、信访、文化等职能向社区延伸。二要努力规范施政行为。要健全政府依法决策程序，对重大决策和重大经济行为实行法律咨询和专家论证，健全重大问题集体决策、社会公示和听证、决策责任制度，提高政府行为的法制化、规范化水平。要加强行政执法监督，认真做好行政复议应诉工作。要全面执行行政执法责任制、过错追究制，严格执行《国家行政许可法》，认真清理现有的行政许可规定和行政许可实施机构，规范行政许可程序，减少行政干预，提高依法行政水平。要加强政务规范化建设，健全政府工作规则，规范政务工作程序，促进政府工作高效规范运转。三要积极推进法制建设。要把依法行政纳入经济社会发展的总体规划和部署，列入重要议事日程，摆上重要工作位置。各级领导干部要加强对依法行政工作的领导和监督，全面落实依法行政工作的各项举措；要带头学法、守法，增强法律意识和法制观念，把政府权力严格限制在法律范围之内，维护法制统一，防止权力滥用；要进一步运用法律手段管理经济和社会事务 ，依法决策和依法行政，努力提高领导能力和水平。要进一步加强民主法制建设，自觉接受人大的法律监督和政协的民主监督，严格执行人大的决议、决定和审议意见，积极听取政协的意见和建议，认真办理人大代表意见和政协提案，切实把政府工作纳入民主化、法制化轨道。

 各位代表，历史已经翻开了崭新的一页，展望未来，前途光明，任重道远，我们要以改革 为动力，以创新为先导，以发展为主题，始终保持蓬勃朝气、昂扬锐气和浩然正气，在市委的领导下，在市人大、市政协的监督、支持、帮助下，竭尽全力加快经济发展，不断推进社会全面进步，为加快老工业基地调整改造和全面建设小康社会做出新的贡献。
